# Supplementary material for: High fructose exposure modifies the amount of adipocyte-secreted microRNAs into extracellular vesicles in supernatants and plasma
Source: PeerJ. 2021 May 19;9:e11305. doi: 10.7717/peerj.11305 (PMC8140597; doi:10.7717/peerj.11305)
Supplement: Supplemental Information 2 [file peerj-09-11305-s002.docx]

**Oil red staining**

For Red Oil staining, 3T3-L1 adipocytes exposed to fructose were fixed with 10% (w/v) formaldehyde for 30 min, PBS washed prior to staining with Oil Red O staining solution. The 3T3-L1 adipocytes were stained with Oil Red O staining solution (Sigma-Aldrich) (1.2mg/mL Oil Red O dye/60% isopropanol solution) for ten minutes at room temperature. The cells were washed with ethanol and PBS to remove Excess Oil Red O dye and photographed at 10X magnification. To quantify intracellular lipid content, total lipids were extracted with isopropyl alcohol and evaluated by spectrometry at 510 nm. Data were normalized to the control according to a previously reported technique (Garcia-Macedo et al., 2008).

**Pparg, Glut4 and Cebpa determination by RT-qPCR**

*Pparg*, *Glut4* and *Cebpa* expression were determined using two-step RT-qPCR in combination with TaqMan probes: *Cebpa*: Left 5’ cgctggtgatcaaacaagag 3’, Right 5’ ggtggctggtaggggaag 3’, Probe: #101; *Glut4*: Left 5’ gacggacactccatctgttg 3’, Right 5’ gccacgatggagacatagc 3’, Probe: #5; *Pparg*: Left 5’ gaaagacaacggacaaatcacc 3’, Right 5’ gggggtgatatgtttgaacttg 3’ Probe: #7; *Gapdh:* Left 5’ aagagggatgctgcccttac 3’, Right 5’ ccattttgtctacgggacga 3’, Probe: #33. Each RT-reaction used 500 ng of RNA using the QuantiNova Reverse Transcription Kit (Qiagen; Hilden, Germany). The RT reaction program and PCR cycling conditions were as we previously reported (Brianza-Padilla et al., 2016). mRNA relative concentrations were normalized with Ct values of *Gapdh*, and values were calculated using 2^−ΔΔCt^ and 2^−ΔCt^ formulas.

**Western blot analysis**

The 3T3-L1 cells were homogenized in lysis buffer (10 mM HEPES, 0.2% Triton X-100, 50 mM NaCl, 0.5 mM sucrose, 0.1 mM EDTA, protease, and phosphatase inhibitors) and the homogenate centrifuged at 10,000 rpm for 10 minutes, at 4°C. The homogenates were used for the determination of total protein concentration by the Bradford method using bovine serum albumin as the standard (HA & LEE, 2005). Proteins 50 μg were resolved by SDS-PAGE and electrotransferred onto a polyvinylidene fluoride membrane (Millipore Corp., Bedford, MA). The following antibodies from Santa Cruz Biotechnology (Santa Cruz Biotechnology, Inc. CA, USA) were used: Glucose transporter type 4 (GLUT4) antibody: sc-7273 E-8 (dilution 1:1000); Peroxisome Proliferator Activated Receptor Gamma (PPARγ) antibody: SC-7273 (dilution 1:1000). Detection of primary antibody was carried out with a horseradish peroxidase–conjugated secondary antibody and enhanced chemiluminescence reagents (Clarity Western ECL Substrate, Bio-Rad). Positive immunoreactive bands were quantified using a Kodak Electrophoresis Documentation and Analysis System 290 (EDAS 290). For loading controls, Coomassie Blue R-250 (Bio-Rad, Hercules, CA) staining was used. The protein expression was expressed as the ratio of the protein interest test sample to loading control in arbitrary units (a.u.).

**Pathway analysis**

The DIANA miRPath web-based bioinformatics program (<http://snf-515788.vm.okeanos.grnet.gr/>) was used to determine pathway enrichment for miRNAs found to be altered by fructose exposure.
