# Supplementary figures and images for: High fructose exposure modifies the amount of adipocyte-secreted microRNAs into extracellular vesicles in supernatants and plasma
Source: PeerJ. 2021 May 19;9:e11305. doi: 10.7717/peerj.11305 (PMC8140597; doi:10.7717/peerj.11305)

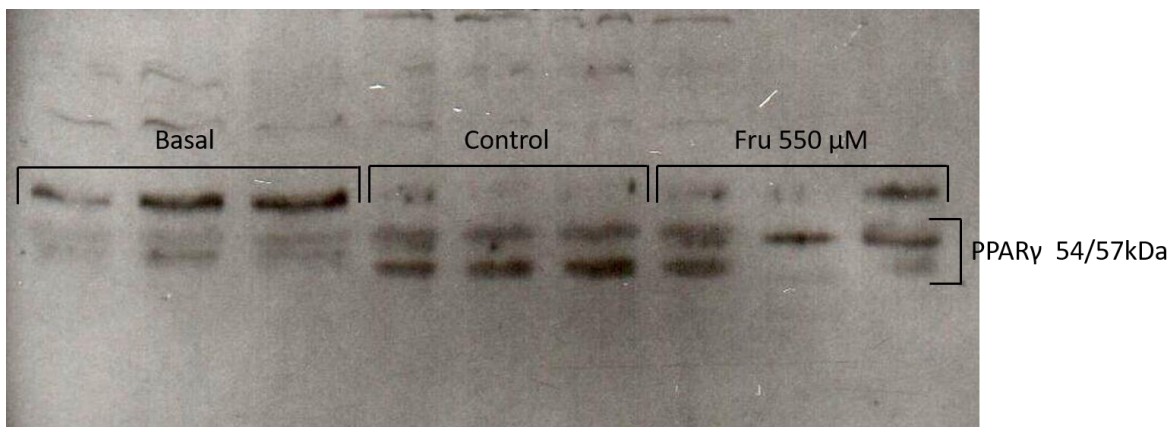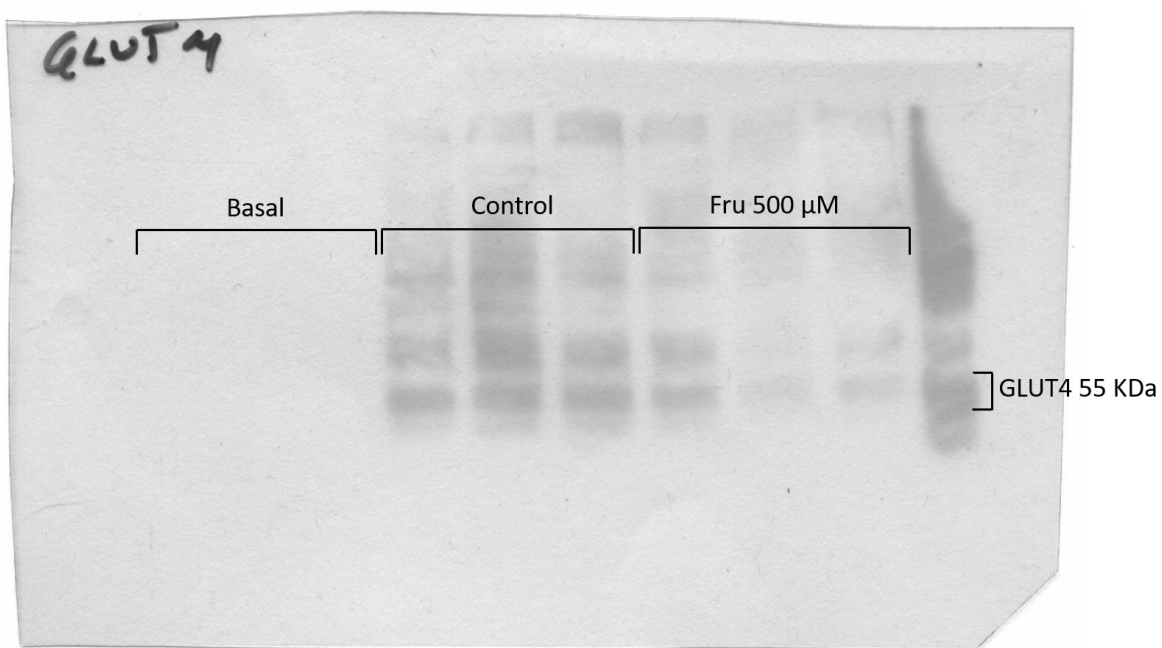

Supplement: Supplemental Information 3 — Western blot of protein of PPARg and GLUT4 from 3T3-L1 cells exposed to fructose. [file peerj-09-11305-s003.pdf]

ANXA2

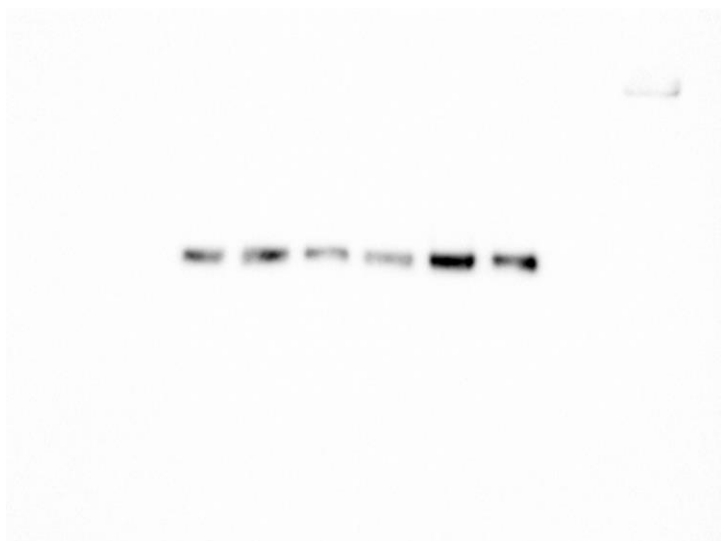

CD63

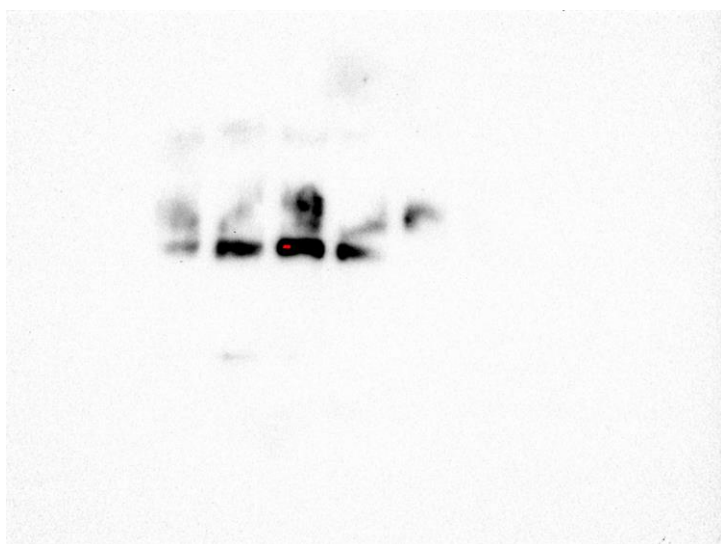

CD81

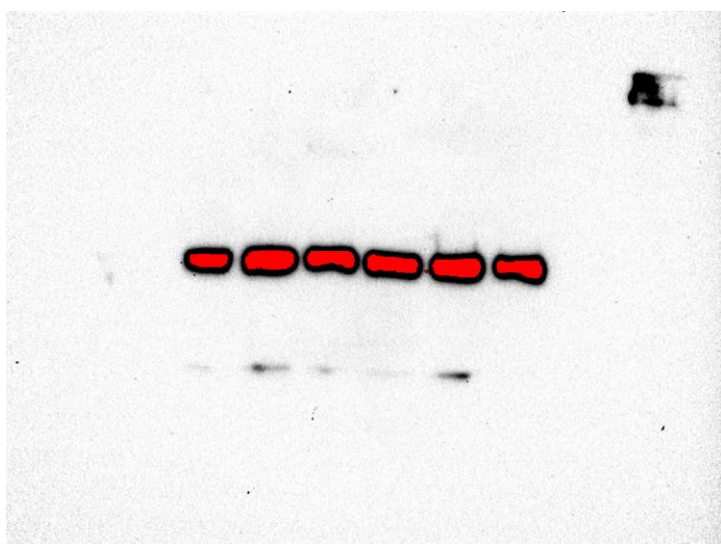

Supplement: Supplemental Information 4 — Western blot of protein of CD63, CD81 and ANXA2 from EVs of supernatants of 3T3-L1 cell exposed to fructose. [file peerj-09-11305-s004.pdf]

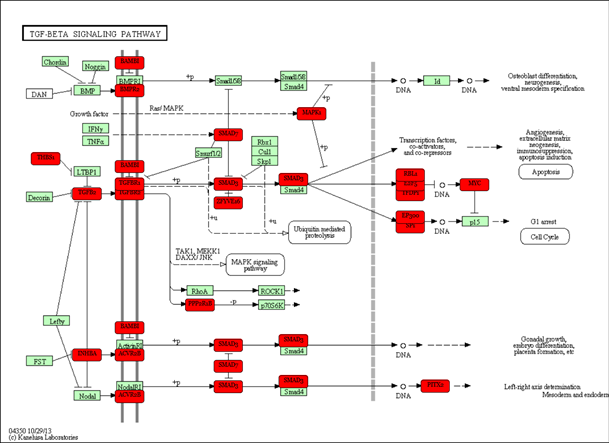

Supplement: Supplemental Information 6 — Regulation of genes predicted using miRPath in the TGF-β signaling pathway targeted by select adipocyte miRNAs. Genes targeted are highlighted with a bolded red box. [file peerj-09-11305-s006.png]

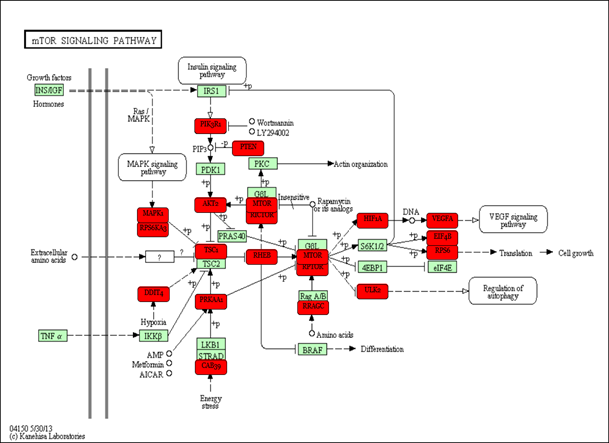

Supplement: Supplemental Information 7 — Regulation of genes predicted using miRPath in the mTOR signaling pathway targeted by select adipocyte miRNAs. Genes targeted are highlighted with a bolded red box. [file peerj-09-11305-s007.png]
