# Supplementary material for: High fructose exposure modifies the amount of adipocyte-secreted microRNAs into extracellular vesicles in supernatants and plasma
Source: PeerJ. 2021 May 19;9:e11305. doi: 10.7717/peerj.11305 (PMC8140597; doi:10.7717/peerj.11305)
Supplement: Supplemental Information 8 [file peerj-09-11305-s008.docx]

| **EVs plasma** | **Triglycerides** | **HDL-C** | **Glucose** | **Leptin** |
| --- | --- | --- | --- | --- |
| miR-21-5p | rho= -0.2887  *p*= 0.2170 | rho= 0.06549  *p*= 0.7839 | rho= -0.1398  *p*= 0.5565 | rho= -0.3922  *p*= 0.1205 |
| miR-223-3p | rho= -0.4088  *p*= 0.0823 | rho= 0.08783  *p*= 0.7207 | rho= -0.3491  *p*= 0.1429 | rho= -0.3382  *p*= 0.2001 |
| miR-450a-5p | rho= 0.04812  *p*= 0.8403 | rho= -0.1340  *p*= 0.5733 | rho= -0.0421  *p*= 0.8601 | rho= -0.2990  *p*= 0.2430 |
| miR-140-5p | rho= -0.4254  *p*= 0.0784 | rho= 0.2885  *p*= 0.2456 | rho= -0.2643  *p*= 0.2892 | rho= -0.3870  *p*= 0.1388 |
| miR-146b-5p | rho= -0.2782  *p*= 0.2350 | rho= 0.1091  *p*= 0.6469 | rho= -0.1805  *p*= 0.4465 | rho= -0.5049  *p*= 0.0408 |
| miR-143-5p | rho= 0.7098  *p*= 0.0005 | rho= -0.4577  *p*= 0.0425 | rho= 0.4256  *p*= 0.0614 | rho= 0.4657  *p*= 0.0615 |
| miR-342-3p | rho= -0.5931  *p*= 0.0137 | rho= 0.3681  *p*= 0.1459 | rho= -0.4240  *p*= 0.0913 | rho= -0.4066  *p*= 0.1505 |
| miR-148a-5p | rho= 0.1279  *p*= 0.5911 | rho= -0.1694  *p*= 0.4752 | rho= 0.6657  *p*= 0.0014 | rho= 0.2502  *p*= 0.3303 |
