# Supplementary material for: High fructose exposure modifies the amount of adipocyte-secreted microRNAs into extracellular vesicles in supernatants and plasma
Source: PeerJ. 2021 May 19;9:e11305. doi: 10.7717/peerj.11305 (PMC8140597; doi:10.7717/peerj.11305)
Supplement: Supplemental Information 9 — Differences were tested by the Mann–Whitney U test. Data are presented as means ± SE. [file peerj-09-11305-s009.docx]

|  | **Control** | **Fructose** | **P-value** |
| --- | --- | --- | --- |
| Glucose (mg/dL) | 101.0 ± 2.83 | 111.9 ± 3.68 | 0.0369 |
| Triglycerides (mg/dL) | 53.44 ± 3.06 | 114.4 ± 21.61 | 0.0052 |
| TotalCholesterol (mg/dL) | 53.21 ± 2.34 | 52.12 ± 2.75 | 0.9296 |
| HDL Cholesterol (mg/dL) | 42.54 ± 1.70 | 39.86 ± 2.28 | 0.4478 |

.
