# Supplementary material for: High fructose exposure modifies the amount of adipocyte-secreted microRNAs into extracellular vesicles in supernatants and plasma
Source: PeerJ. 2021 May 19;9:e11305. doi: 10.7717/peerj.11305 (PMC8140597; doi:10.7717/peerj.11305)
Supplement: Supplemental Information 10 — miRNAs expression was determined by RT-qPCR using cel-miR-39 as a reference for the 2−ΔCt method. Differences were tested by unpaired t-test with Welch’s correction. Data are presented as means ± SE. [file peerj-09-11305-s010.docx]

|  | **Control** | **Ct Control** | **Fructose** | **CtFructose** | **Foldchange** | **P-value** | **Ct P-value** |
| --- | --- | --- | --- | --- | --- | --- | --- |
| miR-223-3p | 0.7595 ± 0.0839 | 21.13 ± 0.2221 | 0.8479 ± 0.0907 | 21.40 ± 0.1952 | 1.11 | 0.4805 | 0.3742 |
| miR-21-5p | 0.1896 ± 0.0188 | 23.42 ± 0.2373 | 0.2123 ± 0.0201 | 23.73 ± 0.1975 | 1.11 | 0.4188 | 0.3152 |
| miR-140-5p | 0.1166 ± 0.0069 | 24.06 ± 0.2013 | 0.1271 ± 0.0097 | 24.19 ± 0.1692 | 1.09 | 0.3862 | 0.6410 |
| miR-342-3p | 0.0309 ± 0.0032 | 25.76 ± 0.1620 | 0.0299 ± 0.0023 | 26.00 ± 0.1161 | -1.03 | 0.8079 | 0.2519 |
| miR-450a-5p | 0.0164 ± 0.0013 | 27.02 ± 0.2354 | 0.0230 ± 0.0027 | 27.07 ± 0.1685 | 1.40 | 0.0376 | 0.8569 |
| miR-143-5p | 0.0092 ± 0.0004 | 27.64 ± 0.1302 | 0.0115 ± 0.0010 | 27.78 ± 0.0870 | 1.25 | 0.0310 | 0.3678 |
| miR-146b-5p | 0.0055 ± 0.0005 | 28.29 ± 0.2252 | 0.0058 ± 0.0006 | 28.18 ± 0.3005 | 1.05 | 0.6848 | 0.7754 |
| miR-148a-5p | 1.522e^-5^ ± 1.984e^-6^ | 36.90 ± 0.2157 | 1.593e^-5^ ± 2.108e^-6^ | 37.07 ± 0.2545 | 1.04 | 0.8092 | 0.6089 |
