# Supplementary material for: High fructose exposure modifies the amount of adipocyte-secreted microRNAs into extracellular vesicles in supernatants and plasma
Source: PeerJ. 2021 May 19;9:e11305. doi: 10.7717/peerj.11305 (PMC8140597; doi:10.7717/peerj.11305)
Supplement: Supplemental Information 11 [file peerj-09-11305-s011.docx]

| **miRNA** | **Pathway** | **Gene targets within the pathway** |
| --- | --- | --- |
| miR-140-5p | TGFβ signaling | *TGFBR1, THBS1, SMAD3, INHBA, TGFB1, ZFYVE16, PPP2CB* |
| miR-143-5p | TGFβ signaling | *PPP2CA* |
| miR-146b-5p | TGFβ signaling | *THBS1, RBL1* |
| miR-223-3p | TGFβ signaling | *ACVR2A, SMAD1, RPS6KB1* |
| miR-342-3p | TGFβ signaling | *SMAD2, THBS1, ACVR2B, EP300, SMAD1, BMPR2* |
| miR-140-5p | mTOR signaling | *PIK3R3, HIF1A, EIF4B, RPS6KA3, VEGFA, CAB39, RRAGC, ULK2* |
| miR-143-5p | mTOR signaling | *PIK3R2, RICTOR* |
| miR-146b-5p | mTOR signaling | *RPS6, RHEB, AKT2, PRKAA1, ULK1* |
| miR-223-3p | mTOR signaling | *DDIT4, ULK2, RPS6KB1* |
| miR-342-3p | mTOR signaling | *PRKAA2, EIF4B, RPS6KB1* |
